# Novel Electrospun PVA-PVP-PAAm/TiO_2_ Nanofibers with Enhanced Optoelectrical, Antioxidant and Antibacterial Performances

**DOI:** 10.3390/polym17182487

**Published:** 2025-09-15

**Authors:** Maher Hassan Rasheed, Mohanad H. Mousa, Qasim Shakir Kadhim, Najmeddine Abdelmoula, Ali Khalfallah, Zohra Benzarti

**Affiliations:** 1 Department of Science, College of Basic Education, University of Babylon, Babylon 51002, Iraqbasic.qasim.shakir@uobabylon.edu.iq (Q.S.K.); 2 Shatrah Technical Institute, Southern Technology University (STU), Basra 61004, Iraq; mohanad.mousa@stu.edu.iq; 3 Laboratory of Multifunctional Materials and Applications (LaMMA), Faculty of Sciences of Sfax, University of Sfax, BP 1171, Sfax 3000, Tunisia; 4 CEMMPRE, ARISE, Department of Mechanical Engineering, University of Coimbra, 3030-788 Coimbra, Portugal

**Keywords:** PVA-PVP-PAAm/TiO_2_ nanofibers, electrospinning, optical properties, electrical conductivity, antibacterial activity, DPPH radical scavenging

## Abstract

Electrospun nanofibers have emerged as a versatile platform for developing advanced materials with diverse applications, owing to their high surface-area-to-volume ratio and tunable properties. The incorporation of metal oxide nanoparticles, such as titanium dioxide (TiO_2_), has proven effective in further enhancing the functional performance of these materials, particularly in optoelectrical, antibacterial, and antioxidant domains. This study presents the first report of electrospun multifunctional nanofibers from a ternary blend of polyvinyl alcohol (PVA), polyvinylpyrrolidone (PVP), and polyacrylamide (PAAm) blended with TiO_2_ nanoparticles at 0, 1, 3, and 5 wt.%. The objective was to develop nanocomposites with enhanced structural, optical, electrical, antibacterial, and antioxidant properties for applications in environmental, biomedical, and industrial fields. The nanofibers were characterized using X-ray diffraction (XRD), field emission scanning electron microscopy (FESEM), Fourier-transform infrared spectroscopy (FTIR), UV–visible spectrophotometry, and DC electrical conductivity tests. Antibacterial efficacy was assessed against Escherichia coli and Staphylococcus aureus via the Kirby–Bauer disk diffusion method, while antioxidant activity was evaluated using the DPPH radical scavenging assay. Results demonstrated that TiO_2_ incorporation increased nanofiber diameters (21.5–35.1 nm), enhanced crystallinity, and introduced Ti–O bonding, confirming successful nanoparticle integration. Optically, the nanocomposites exhibited reduced band gaps (from 3.575 eV to 3.320 eV) and increased refractive indices with higher TiO_2_ nanoparticle content, highlighting their potential for advanced optoelectronic devices such as UV sensors and transparent electrodes. Electrically, conductivity improved due to increased charge carrier mobility and conductive pathways, making them suitable for flexible electronics and sensing applications. The 5 wt.% TiO_2_-doped nanofibers demonstrated superior antibacterial activity, particularly against *E. coli* (18.2 mm inhibition zone), and antioxidant performance comparable to ascorbic acid (95.32% DPPH inhibition), showcasing their relevance for biomedical applications like wound dressings and food packaging. These findings highlight the potential of PVA-PVP-PAAm/TiO_2_ nanofibers as useful materials for moisture sensors, antibacterial agents, and antioxidants, advancing applications in medical devices and environmental technologies.

## 1. Introduction

Electrospinning is a foundational technique for nanofiber fabrication, valued for its simplicity, versatility, and compatibility with a wide range of natural and synthetic polymers. This method produces nanofibers with diameters ranging from tens of nanometers to several micrometers [1,2]. A key factor in electrospinning is the solvent evaporation rate, which significantly influences fiber morphology and quality. Optimal results are achieved by dissolving polymers in solvents or solvent mixtures with high dielectric constants, while blending polymers with diverse structures and functionalities can further enhance nanofiber properties [3,4,5]. Despite these advances, achieving multifunctional nanofibers with tailored properties for specific applications remains challenging.

Polymers are essential materials, valued for their affordability, versatility, and robust chemical, physical, and optical properties, making them ubiquitous across industries. Nanocomposites have gained prominence due to their superior properties compared to their individual constituents. These lightweight, corrosion-resistant, and easily processed materials rely on the precise integration of nanoscale reinforcement phases within a polymer matrix [6]. Unlike conventional composites, nanocomposites incorporate nanoscale fillers, such as zinc or tin oxide nanoparticles, which significantly enhance their mechanical, electrical, and optical characteristics. The matrix, reinforcement, and interfacial adhesion mechanisms are pivotal to their functional performance [7,8,9].

Polyvinyl alcohol (PVA) is a versatile polymer known for its excellent insulating properties, tunable electrical and optical characteristics, and high storage capacity, making it widely used in various applications [10,11]. Polyvinylpyrrolidone (PVP), an amorphous, water-soluble polymer, offers exceptional biocompatibility, thermal stability, and pH buffering, making it ideal for pharmaceutical and nanofiber applications [12]. Polyacrylamide (PAAm), another water-soluble polymer, is valued for its high molecular weight, non-toxicity, and industrial utility as a flocculant. Its derivatives and copolymers are applied in mining and water treatment when blended with polymers such as polyethylene glycol (PEG), PVA, or carboxymethyl cellulose (CMC) [13,14]. PVA–PVP blends, in particular, exhibit strong interactions with inorganic additives, serving as hydrophilic binders and coating agents in composite fabrication [15].

Titanium dioxide (TiO_2_), non-toxic ceramic nanoparticles, are renowned for their physicochemical properties, including antibacterial efficacy, and for their applications in electronics, medicine, and environmental remediation [16]. Available in anatase, rutile, and brookite crystalline phases, TiO_2_ nanoparticles enhance the functionality of polymer-based materials [16,17]. Although polymers are widely used, their limitations in biological, electrical, and antimicrobial applications can be addressed by incorporating nanoparticles like TiO_2_, thereby expanding their scope [18,19].

In this study, we introduce a novel composite by developing PVA-PVP-PAAm/TiO_2_ nanocomposites, leveraging the synergistic strengths of these materials to develop multifunctional nanofibers. PVA provides excellent film-forming capabilities and good electrical insulating properties, making it a foundational component for the nanofiber matrix and contributing to the overall structural integrity and electrical characteristics [20,21,22]. PVP enhances the electrospinning process due to its high water solubility and excellent biocompatibility, which are crucial for biomedical applications [23,24]. Its ability to form hydrogen bonds also facilitates the dispersion of nanoparticles and improves the blend’s homogeneity. PAAm contributes to the hydrophilic nature of the composite and can improve the structural integrity and stability of the nanofibers [25,26]. Its presence is particularly beneficial for applications requiring water absorption or interaction with biological environments. TiO_2_ nanoparticles are incorporated to impart enhanced optoelectrical, antibacterial, and antioxidant properties. TiO_2_ nanoparticles are also known for their photocatalytic activity, which contributes to their antibacterial efficacy, as well as their semiconductor properties, which influence the optical band gap and electrical conductivity of the composite. The synergistic combination of these three polymers with TiO_2_ nanoparticles is designed to create a robust and versatile material with tailored properties for specific applications. Unlike previous studies, this work distinguishes itself by integrating three polymers (PVA-PVP-PAAm) with TiO_2_ nanoparticles to fabricate nanocomposite films exhibiting enhanced structural integrity, optical and electrical properties, antibacterial performance, and antioxidant activity. This work emphasizes the synergistic interactions of these components to unlock multifunctional properties suited for biomedical technologies, environmental applications, and advanced material systems.

These properties were systematically characterized using advanced techniques, including field emission scanning electron microscopy (FESEM), Fourier-transform infrared spectroscopy (FTIR), X-ray diffraction (XRD), UV–Visible spectrophotometry, DC electrical testing, and antibacterial and antioxidant assays. The resulting nanocomposites demonstrate significant potential as moisture sensors, bio-based antibacterial agents, and antioxidants, addressing significant needs in environmental, biomedical, and industrial applications. This blend, elaborated using the electrospinning process, represents a significant advancement in the design of high-performance nanocomposites.

## 2. Materials and Methods

### 2.1. Materials

Polyvinyl acetate (C_2_H_4_O)_n_, with a molecular weight of 85,000 g/mol, was supplied by Himedia, India. Polyvinylpyrrolidone (C_6_H_9_ON)_n_, with a molecular weight of 1,350,000 g/mol, was obtained from Glentham Life Sciences Limited. Polyacrylamide (PAAm) (C_3_H_5_NO)_n_, with a molecular weight of 5 × 10^5^ g/mol and an ultra-high purity of 99.99%, was provided by British Pharma (BDH). Titanium dioxide (TiO_2_) nanoparticles (10–35 nm, 47.867 g/mol, 99.6% purity) were purchased from Sigma-Aldrich (https://www.sigmaaldrich.com, accessed on 5 September 2025). Ultrapure water (UPW) was used throughout the experiments. Additional reagents included dimethyl sulfoxide (DMSO), penicillin, streptomycin, phosphate-buffered saline (PBS), and Dulbecco’s Modified Eagle Medium (DMEM).

### 2.2. Preparation of PVA-PVP-PAAm/TiO_2_ Nanoparticles

**Figure 1:**
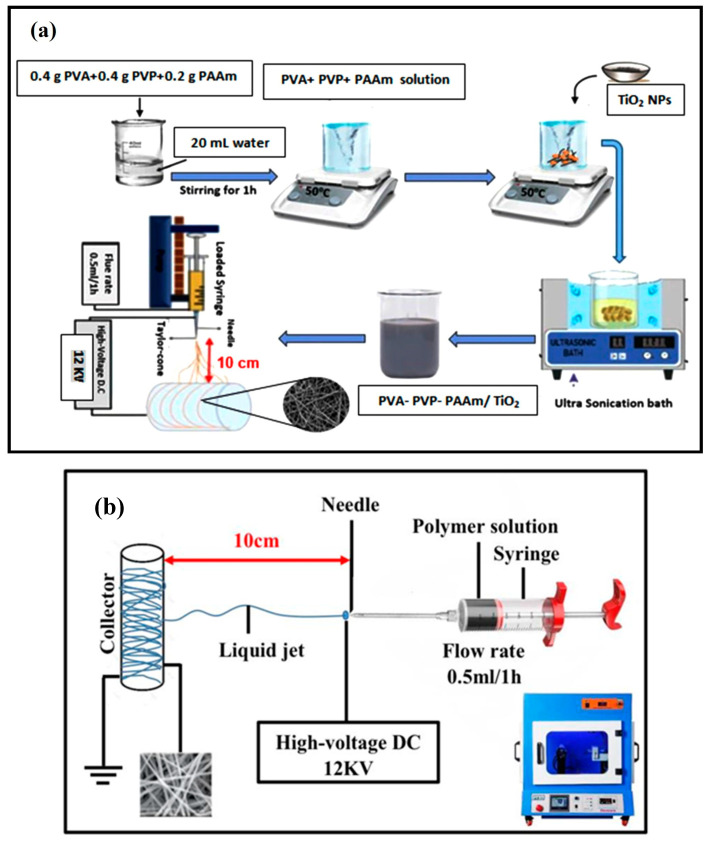
(**a**) Schematic representation of the preparation and electrospinning process for PVA/PVP/PAAm nanofibers incorporated with TiO_2_ nanoparticles and (**b**) schematic diagram of the electrospinning setup used to fabricate nanofibers.

PVA and PVP were first prepared as separate solutions. The first solution was obtained by dissolving 0.4 g of PVA in 20 mL of UPW under magnetic stirring at 500 rpm for 1 h at room temperature. The second solution was similarly prepared by dissolving 0.4 g of PVP in 20 mL of UPW, using the same stirring conditions. The two solutions were then combined and stirred together for an additional hour to obtain a homogeneous aqueous mixture. Subsequently, 0.2 g of PAAm was added to the blended solution, and stirring continued until complete dissolution. Using this PVA-PVP-PAAm polymer matrix, four samples were prepared by doping with TiO_2_ nanoparticles at different contents (0 wt.%, 1 wt.%, 3 wt.%, and 5 wt.%). All samples were prepared using the same solvent, without any heating during the doping process. The resulting mixtures were then subjected to ultrasonication for 2 min at 80 °C to ensure uniform dispersion of the nanoparticles. These final preparations were suitable for subsequent nanofiber fabrication. Figure 1a illustrates the step-by-step preparation process of a composite solution containing PVA, PVP, PAAm, and TiO_2_ nanoparticles, culminating in the formation of nanofiber mats via a high-voltage setup.

Electrospun nanofiber mats were fabricated using an electrospinning machine (Model: ES-106, Nanolab Instruments Co., Subang Jaya, Malaysia) using PVA-PVP-PAAm polymer solution adding various contents of TiO_2_ nanoparticles. To prepare the glass substrates (2.5 × 3 cm^2^) for use in optical characteristics tests, they were first wrapped in aluminum foil, cleaned several times with ethanol and distilled water, and then affixed using adhesive tape. The prepared polymer solution was loaded into a 5 mL syringe equipped with a metal capillary needle. Electrospinning was performed at a flow rate of 0.5 mL/h, with the needle rotating at 200 rpm and an applied voltage of 12 kV. The distance between the needle and the collector drum was maintained at 10 cm, as illustrated in Figure 1b.

The fabricated samples were designated as follows:

- B: PVA-PVP-PAAm blend
- BT1: PVA-PVP-PAAm with 1 wt.% TiO_2_
- BT3: PVA-PVP-PAAm with 3 wt.% TiO_2_
- BT5: PVA-PVP-PAAm with 5 wt.% TiO_2_

### 2.3. Characterization of the PVA-PVP-PAAm/TiO_2_ Nanofibers

The crystalline nature of the compounds present in the nanofiber was characterized using X-ray diffraction (XRD) (Philips X’Pert High Score, PANalytical, Almelo, The Netherlands) with Cu Kα radiation (λ = 0.1542 nm), operated at 40 kV, and scanned over a 2θ range of 10–80°. The surface morphology of the nanofibers was characterized using field emission scanning electron microscopy (FESEM) (Tescan MIRA III, Brno, Czech Republic). The fiber diameter was measured using FESEM micrographs and ImageJ free software, version 1.54p 17 February 2025. The chemical composition of the nanofiber mats at room temperature was analyzed by Fourier-transform infrared spectroscopy (FTIR) (Thermo Nicolet Nexus 670 FTIR, HEADQUARTERS, 12 Colton Road, East Lyme, CT 06333, USA) within a spectral range of 4000–400 cm^−1^. The FTIR analysis was conducted in Attenuated Total Reflection (ATR) mode, directly on electrospun nanofiber mats, eliminating the need for KBr pellet preparation. Optical properties were investigated using a UV–Visible spectrophotometer, Shimadzu^TM^ UV-1800A, Nishinokyo-Kuwabara-cho, Nakagyo-ku, Kyoto 604-8511, Japan) to record transmission spectra in the 200–800 nm range. These measurements were performed on the electrospun nanofiber mats deposited on glass substrates. For electrical characterization, a DC Keithley 2450 Digital Multimeter (Tektronix UK Ltd. The Capitol Building, Oldbury, Bracknell Berkshire RG12 8FZ, UK) and Source Measure Unit (SMU) were used. The read pulse was set to 200 μs, and the time between read and write pulses was 700 μs.

Antimicrobial activity was assessed using the Kirby–Bauer disk diffusion method, following the protocol described by Humphreys et al. [27]. Antibacterial efficiency was tested against two bacterial strains: *Staphylococcus aureus* (ATCC 29213) and *Escherichia coli* (ATCC 35218).

Antioxidant activity was evaluated based on the DPPH (1,1-diphenyl-2-picrylhydrazyl) free radical scavenging assay. A 0.025 g/L DPPH solution was prepared by dissolving it in methanol. Different nanofiber samples, including PVP/TiO_2_, PAAM/TiO_2_, and PVA-PVP-PAAm/TiO_2_, each incorporated with 5 wt.% of TiO_2_, were dissolved in DMSO. Ascorbic acid was used as the reference (control) solution. In a 96-well microplate, 5 μL of each sample solution was added to the wells, followed by 195 μL of the DPPH solution. After a 20 min incubation at room temperature, absorbance was measured at 517 nm using a spectrophotometer. DPPH scavenging activity (%) was calculated using the following formula [28]:

(1) $$\mathrm{DPPH}\;\mathrm{Scavenging}\;\mathrm{Activity}\;(\%)=\left(\frac{A_{0}-A_{1}}{A_{0}}\right)\times 100$$

where $A_{0}$ represents the absorbance of the control (ascorbic acid), and $A_{1}$ is the absorbance of the test sample.

## 3. Results

### 3.1. XRD Investigation

**Figure 2:**
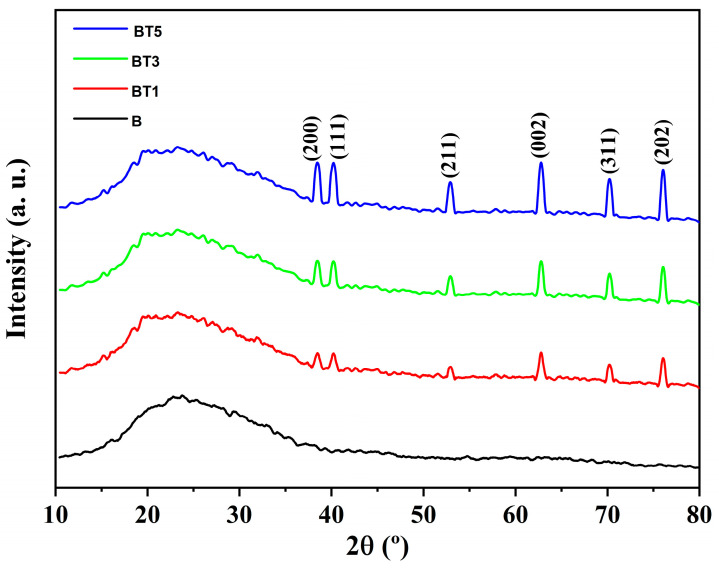
XRD patterns of the PVA-PVP-PAAm/TiO_2_ nanofibers (B, BT1, BT3, and BT5).

To examine the crystalline nature of the compounds present in the nanofibers, XRD patterns were recorded for the PVA-PVP-PAAm polymer blend (B) and its nanocomposites containing 1 wt.% (BT1), 3 wt.% (BT3), and 5 wt.% (BT5) of TiO_2_ nanoparticles, as shown in Figure 2. The characteristic diffraction peaks of PVA, previously reported by Ragab et al. [18], occur at approximately 2θ = (19.32°–23.12°). In this study, the PVA-PVP-PAAm polymer blend exhibits a broad diffraction band, confirming its semi-crystalline nature. This band results from the superposition of individual polymer characteristics, with amorphous scattering likely dominating due to the presence of PVP and PAAm and a potential reduction in PVA crystallinity. With TiO_2_ nanoparticles’ incorporation, additional sharp diffraction peaks emerge in the XRD patterns of the nanocomposites, corresponding to the (110), (200), (111), (211), (002), (311), and (202) crystal planes of TiO_2_. Rutile peaks were observed at 2θ values of approximately 27.43°, 39.18°, 41.23°, 54.31°, 62.75°, 72.41° and 76.52°, respectively, aligning with the tetragonal crystalline structure of TiO_2_ per JCPDS card No. 72-1148. These findings confirm the successful incorporation of TiO_2_ nanoparticles into the polymer matrix, consistent with previous studies, reporting enhanced crystallinity in polymer matrices upon TiO_2_ incorporation [16,17].

### 3.2. Fiber Morphology and Diameter

**Figure 3:**
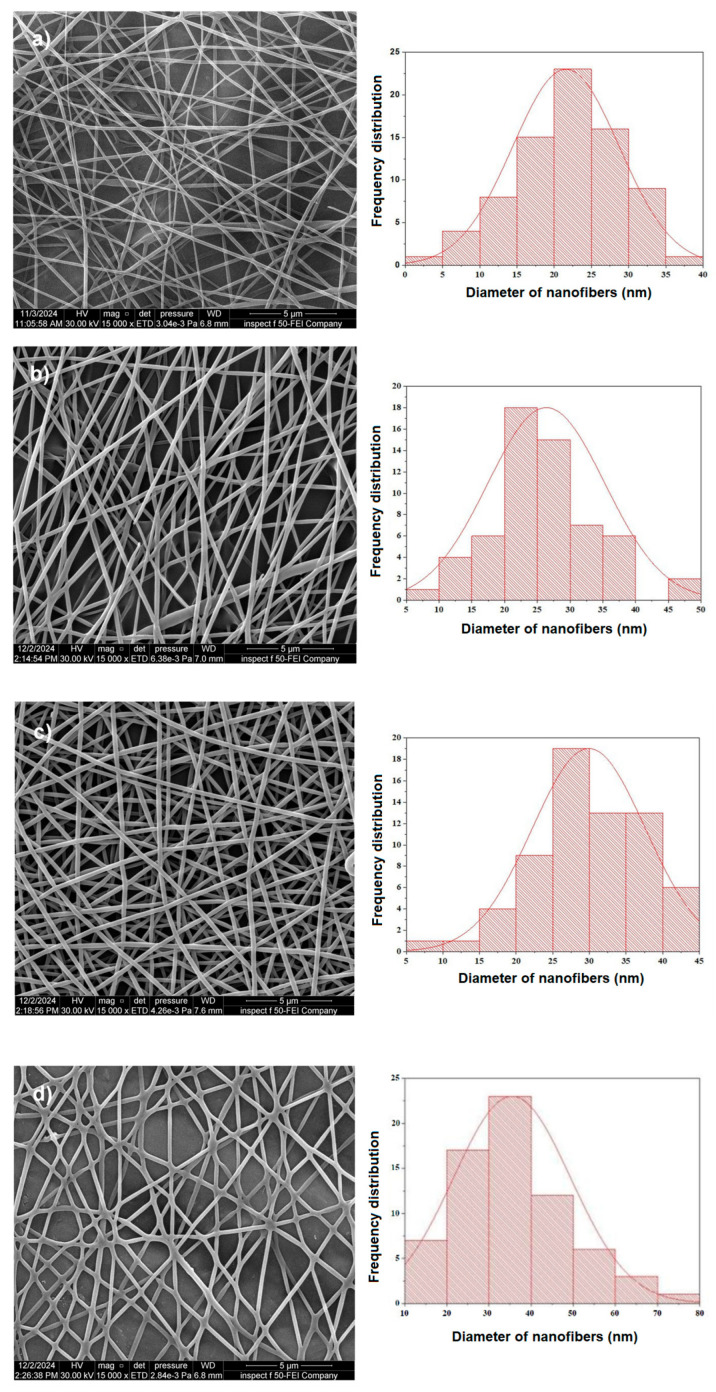
FESEM images of the PVA-PVP-PAAm/TiO_2_ nanofibers: (**a**) B, (**b**) BT1, (**c**) BT3, and (**d**) BT5 with histograms of diameters with a Gaussian distribution.

Figure 3 presents FESEM images of electrospun nanocomposites B, BT1, BT3, and BT5, along with the corresponding histograms of nanofiber diameter distributions. The images recorded at 15,000× *g* (magnification) exhibit a Gaussian distribution. All samples display uniform structures with smooth surfaces, comprising linear and branched nanofibers. The average diameters of nanofibers were measured to be 21.5 nm (B), 26.3 nm (BT1), 30.1 nm (BT3), and 35.1 nm (BT5). The diameter of electrospun nanofibers is governed by the interplay of electrical forces acting during the spinning process. Intensification of these attractive forces can limit fiber elongation and thinning, thereby influencing final fiber dimensions. In this study, a progressive increase in nanofiber diameter was observed with increasing TiO_2_ nanoparticle content, suggesting a direct correlation between nanoparticle loading and fiber diameter. Furthermore, variations in charge density and solution viscosity, both dependent on TiO_2_ content, were found to contribute significantly to the observed differences in nanofiber diameter [5,29].

### 3.3. FTIR Investigation

**Figure 4:**
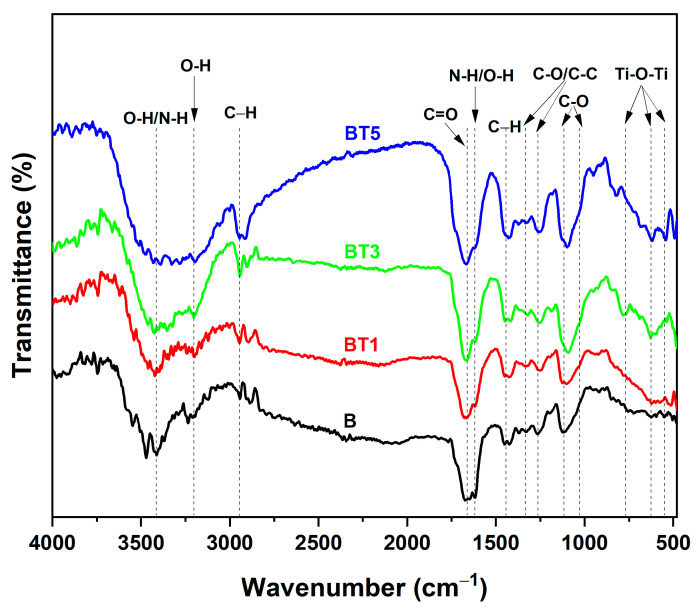
FTIR spectra of the PVA-PVP-PAAm/TiO_2_ nanofibers: B, BT1, BT3, and BT5.

Figure 4 presents the FTIR spectra of the PVA-PVP-PAAm film (B) with varying concentrations of TiO_2_ filler, from 1 wt.% to 5 wt.%. TiO_2_ exhibits characteristic absorption bands that reveal the molecular interactions and structural features of the polymer matrix and incorporated filler. A broad band centered in the 3200–3500 cm^−1^ range is attributed to overlapping O–H stretching of hydroxyl groups from PVA and adsorbed water on TiO_2_, as well as N–H stretching from PAAm, which broadens, shifts slightly, and decreases in intensity with higher TiO_2_ loading due to hydrogen bonding interactions between the polymer functional groups and the nanoparticle surface [30,31,32,33]. Notably, the overlap and broadening of these bands indicate hydrogen bonding interactions among the blend constituents, enhancing the miscibility of the three polymers [29,34]. Peaks at 2890–2950 cm^−1^ correspond to symmetric and asymmetric C–H stretching vibrations from CH2/CH3 groups present in PVA, PVP, and PAAm [35]. With increasing TiO_2_ content, the C–H asymmetric stretching vibrations show minor variations, likely indicating a slight conformational change in the polymer backbone due to the presence of TiO_2_ nanoparticles [36,37]. The peak of 1650 cm^−1^ is assigned to C=O stretching, encompassing the amide I mode from PAAm and carbonyl from PVP [34,38]. Peak around 1620 cm^−1^ corresponds to N–H bending, encompassing amide II from PAAm and O–H bending from the TiO_2_ surface [34]. Further assignments include C–H bending at 1440 cm^−1^ from the polymers, C–N stretching from PVP and C–O/C–C stretching from PVA at 1280–1320 cm^−1^ with minor shifts observed upon TiO_2_ addition and C–O stretching (alkoxy/epoxy) from PVA/PVP at 1020–1100 cm^−1^ [39]. It is noted that C–O stretching vibrations (peaks around 1100–1125 cm^−1^) and methylene (C–H) bending modes (around 1450 cm^−1^) exhibit slight shifts and intensity changes, reflecting modifications in the local chemical environment due to nanoparticle dispersion [34]. With TiO_2_ incorporation, subtle shifts in peak positions and intensity changes are observed. Notably, the band between 550 and 750 cm^−1^ displays Ti–O–Ti stretching and bending modes, with progressive intensity enhancement correlating to higher TiO_2_ content, verifying effective filler dispersion and matrix filler interactions [40]. Interestingly, the decrease in overall transmittance with increasing TiO_2_ content indicates enhanced internal scattering and reflection, arising from the higher density of scattering centers introduced by the nanoparticles. As TiO_2_ loading increases, additional polymer–filler interfaces are formed, which further scatter light due to the refractive-index mismatch between the PVA/PVP/PAAm matrix and TiO_2_. This phenomenon may enhance UV-shielding capabilities or alter transparency, depending on the application. The FTIR spectra demonstrate interfacial interactions between the miscible chain architectures and TiO_2_ nanoparticles in the PAAm-PVA-PVP blend. The observed spectral changes highlight the role of TiO_2_ in modifying hydrogen bonding and vibrational modes within the composite, potentially improving its functional properties for advanced applications. These results strongly agreed with previous reports [32,40,41].

### 3.4. UV–Visible Spectroscopy

**Figure 5:**
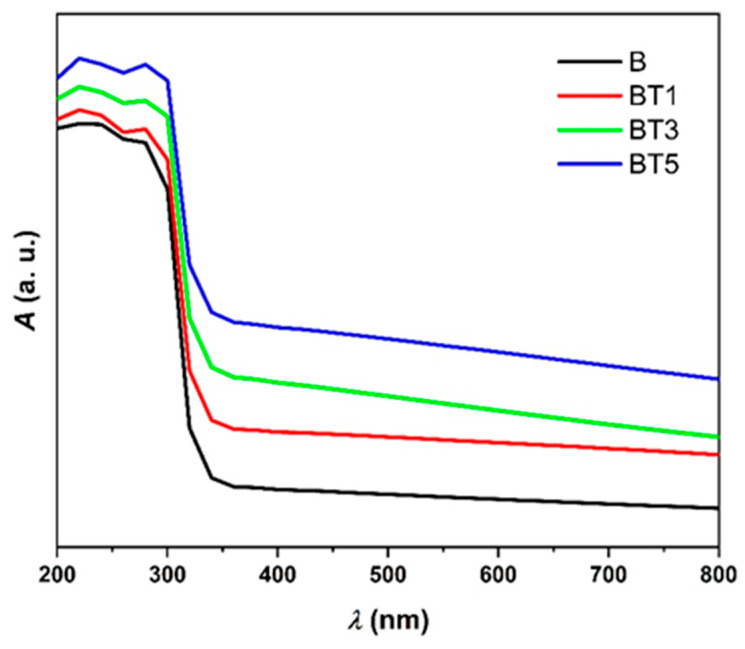
Room temperature UV–visible absorbance spectra (A) of the PVA-PVP-PAAm/TiO_2_ nanofibers (B, TB1, BT3 and BT5) with different TiO_2_ nanoparticle contents.

Figure 5 shows the room temperature UV–visible absorbance spectra of electrospun PVA-PVP-PAAm/TiO_2_ nanofibers. Absorbance spectra for samples B, BT1, BT3 and BT5 were obtained in the 200–800 nm range. The spectra reveal that TiO_2_ nanoparticle incorporation significantly affects the optical properties of nanocomposites. All films exhibit minimal absorbance in the visible range and significant absorbance in the UV region, due to strong π-π* transitions associated with C=C bonds and plasmonic interactions within the PVA-PVP-PAAm blend. This phenomenon can be explained by the fact that photons at longer wavelengths (lower energy) lack sufficient energy to interact with the material’s atoms, resulting in transmission, whereas photons at shorter wavelengths (near the fundamental absorption edge) possess enough energy to induce electron absorption. Increasing TiO_2_ nanoparticle content enhances absorbance, as these particles promote light absorption by free electrons in the material [32,40]. Notably, higher TiO_2_ nanoparticle content induces a redshift in the absorption edge, indicating n → π* transitions and increased conjugation lengths, consistent with literature reports on electron transitions within the bandgap and polymer-nanoparticle interactions [42].

The absorption coefficient (*α* in cm^−1^) was calculated using the following equation [42]:

(2) $$\alpha =2.303\;\frac{A}{t}$$

where A represents the absorbance measured at a given wavelength, and *t* is the thickness of the film in centimeters (e.g., *t*~75 μm for the PVA-PVP-PAAm blend and *t*~80 μm for the PVA-PVP-PAAm/TiO_2_ nanofibers).

**Figure 6:**
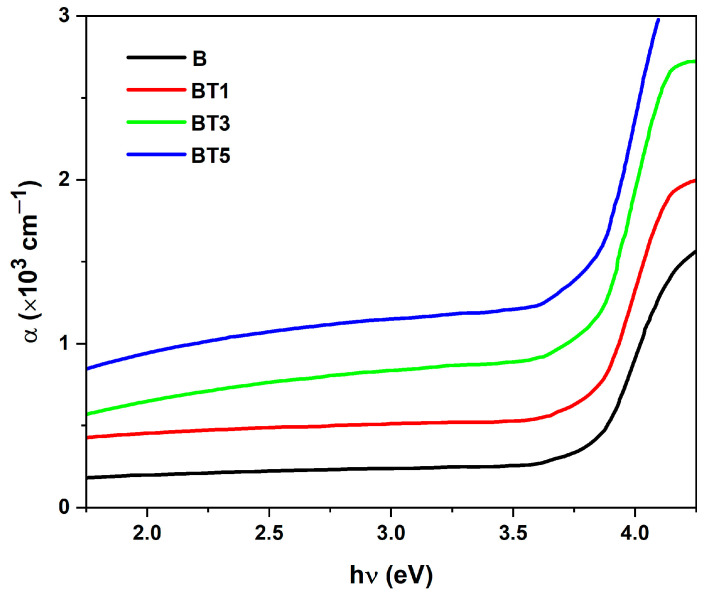
Variation in the absorption coefficient (*α*) as a function of photon energy (*hν*) for PVA-PVP-PAAm blend (B) and PVA-PVP-PAAm/TiO_2_ nanofibers (BT1, BT3 and BT5) with different TiO_2_ nanoparticle contents.

Figure 6 illustrates the variation in absorption coefficient versus photon energy of the PVA-PVP-PAAm/TiO_2_ nanofibers. Electron absorption is less efficient at low energies but becomes particularly pronounced at high energies, suggesting that indirect transitions are more probable when the absorption coefficient is below 10^4^ cm^−1^ [42].

**Figure 7:**
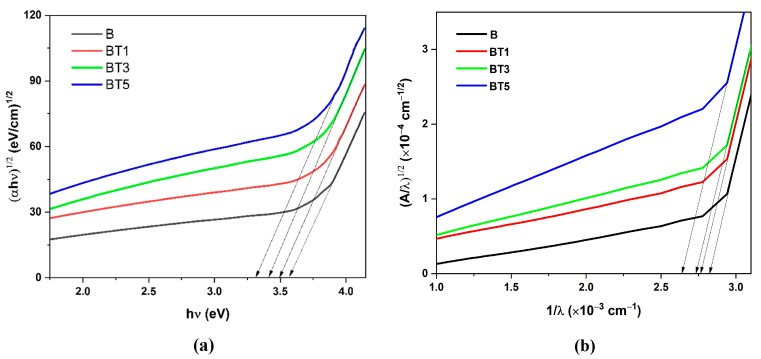
(**a**) Tauc’s plot of $\;{\left(\alpha h\nu \right)}^{\frac{1}{2}}\;$ versus hν, (**b**) ASF plot of (A/*λ*)^1/2^ versus 1/*λ* for the PVA-PVP-PAAm/TiO_2_ nanofibers.

The energy band gap shift was observed by analyzing the absorption edge of the PVA-PVP-PAAm/TiO_2_ samples, influenced by varying degrees of semi-crystallinity in the polymer matrix. The optical bandgap ${E_{g}}^{opt}$ was determined using the Tauc relation [42]:

(3) $$\left(\alpha h\nu {)}^{m}=B(h\nu -{E_{g}}^{opt}\pm E_{ph}\right)$$

where $E_{ph}$ denotes phonon energy, with a negative sign (−), indicating phonon absorption and a positive sign (+), indicating phonon emission. For allowed indirect transitions, *m* = ½. The indirect optical energy gap ($E_{g\;indir.}^{opt}$) was determined by extrapolating the linear portion of the absorption spectrum curve to the photon energy axis, at ${\left(\alpha h\nu \right)}^{1/2}=0$, as shown in Figure 7a. The goal was to determine the depth of the indirect optical energy gap. Table 1 lists the values of $E_{g\;indir.}^{opt}$ for the PVA-PVP-PAAm/TiO_2_ nanofibers. As expected, $E_{g\;indir.}^{opt}$ decreases with increasing TiO_2_ nanoparticle content, as electron transition from the valence band to localized levels and then to the conduction band due to defect states introduced with the forbidden bandgap. Higher TiO_2_ concentrations reduce the energy levels, as reported in Table 1, affecting the electronic structure and optical properties of nanofibers [34,43].

The Absorption Spectra Fitting (ASF) model, independent of film thickness, was also employed to evaluate the indirect optical energy gap $E_{g\;indir.}^{opt}$ [44]. This was determined by extrapolating the linear segment of the (A/*λ*)^1/2^ versus 1/*λ* plot to (A/*λ*)^1/2^ = 0, as depicted in Figure 7b. The optical bandgap was calculated using $E_{ASF}^{opt}=\frac{1240}{\lambda }$. Notably, the $E_{ASF}^{opt}$ values, reported in Table 1, are comparable to those obtained via the Tauc method.

The defects and structural disorder of the polymer matrix are characterized by the Urbach tail energy ($E_{U}$). According to the Urbach model, this band tail follows the empirical relation [45]:

(4) $$\ln(\alpha )=\ln(\alpha_{0})\;+\;\frac{h\nu }{E_{U}}$$

**Figure 8:**
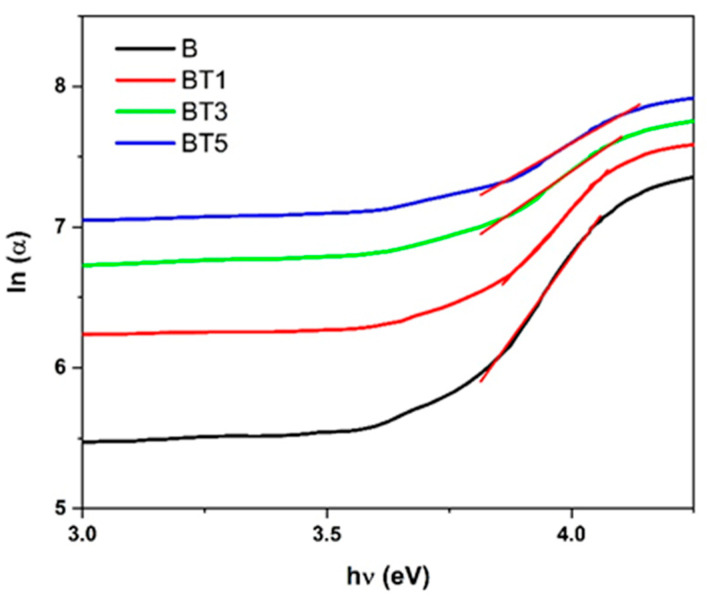
Plot of *ln*(*α*) versus hν for the different nanofiber films. The red lines indicate the linear-fit regions; the inverse of the slope in these regions yields the Urbach energy.

Figure 8 shows the plot of ln(α) versus hν, with the Urbach tail energy ${(E}_{U})$ determined as the inverse of the slope of the linear region.

The $E_{U}$ values for the PVA-PVP-PAAm/TiO_2_ nanofibers, which range from 0.208 eV to 0.507 eV, exhibit a clear increasing trend with higher TiO_2_ nanoparticle content. This increase in $E_{U}$ reflects a broadening of the Urbach tail in the optical absorption spectrum, which is commonly associated with enhanced structural disorder and the formation of localized states within the band gap. The incorporation of TiO_2_ nanoparticles disrupts the regular packing of the polymer chains, introducing defects and microstructural irregularities that elevate the degree of disorder in the nanocomposites. Consequently, the observed rise in $E_{U}$ indicates that the addition of TiO_2_ nanoparticles significantly influences the electronic structure of the polymer blend, leading to a more disordered system.

The refractive index (n) and extinction coefficient (k) were calculated using Equations (5) and (6), respectively [32]:

(5) $$n=\sqrt{\frac{4R-k^{2}}{{\left(R-1\right)}^{2}}}-\frac{\left(R+1\right)}{\left(R-1\right)}$$

(6) $$k=\frac{\alpha \lambda }{4\pi }$$

**Figure 9:**
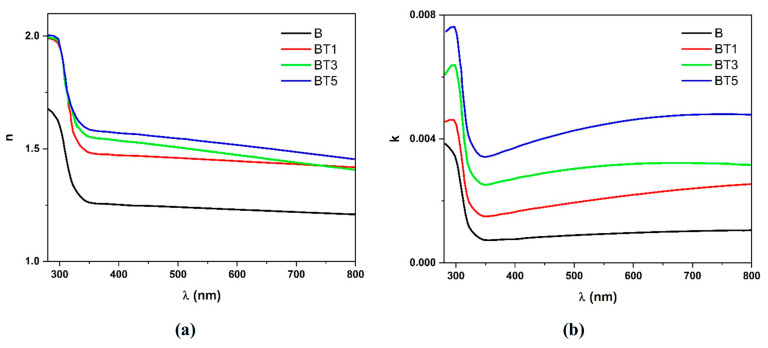
(**a**) Variation in the refractive index (*n*) and (**b**) the extinction coefficient (*k*) in relation to the wavelength (*λ*) of the different PVA-PVP-PAAm/TiO_2_ nanofibers.

The wavelength-dependent refractive index of the PVA-PVP-PAAm/TiO_2_ nanofibers is shown in Figure 9a. It is evident that the refractive index increases with increasing TiO_2_ nanoparticle content. This behavior is attributed to the higher density of nanofibers, leading to enhanced interaction with incident light, which consequently propagates less through the nanocomposite [46]. As a result, the ultraviolet refractivity of the sample increases, raising the refractive index values. The significant refractive index values suggest promising applications in strong optical confinement and enhancement of nonlinear optical intensities, indicating their potential for advanced optoelectronic devices [46].

Figure 9b shows the variation in the extinction coefficient with wavelength for the PVA-PVP-PAAm/TiO_2_ nanofibers. Both optical absorption and photon scattering increase with increasing TiO_2_ nanoparticle content. Consequently, the extinction coefficient increases. This behavior may be associated with the strong absorbance of the nanofiber samples, resulting in higher extinction coefficient values at ultraviolet wavelengths. Furthermore, since the absorption coefficient remains relatively constant across the visible to near-infrared range, the extinction coefficient of the nanofibers increases as the wavelength increases. This behavior aligns with the findings of Alawi and Al-Bermany [34].

The real part of the dielectric constant ($\varepsilon^{\prime }$) and the imaginary component of the dielectric constant ($\varepsilon^{\prime \prime }$) were calculated using Equations (7) and (8) [32]:

(7) $$\varepsilon^{\prime }=n^{2}-k^{2}$$

(8) $$\varepsilon^{\prime \prime }=2nk$$

**Figure 10:**
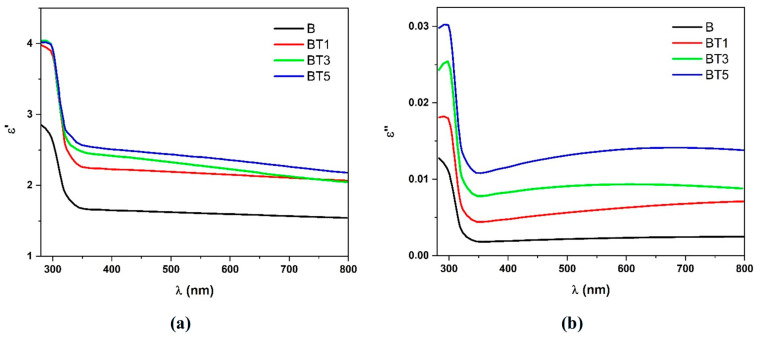
(**a**) Real dielectric constant ($\varepsilon^{\prime }$) and (**b**) imaginary dielectric constant ($\varepsilon^{\prime \prime }$) as a function of the wavelength (*λ*) for the different PVA-PVP-PAAm/TiO_2_ nanofibers.

Figure 10a,b illustrate the variation in the real and imaginary parts of the dielectric constant along with wavelength. An increase in the real and imaginary components is observed with increasing TiO_2_ nanoparticle content, which can be attributed to the decrease in the photon scattering and the increase in the electrical polarization within the nanocomposites [34]. As the wavelength increases, both the real and imaginary parts of the dielectric constant decrease due to the higher absorption coefficient of the nanofibers [40]. Notably, both the real and imaginary components show similar trends with wavelength. However, the real part of the dielectric constant is consistently greater than the imaginary part. This observation might be attributed to the minimal energy dissipation and the enhanced speed of light within the nanocomposites [40]. These results align with previously reported behavior [34]. Interestingly, our dielectric loss values are significantly lower than those reported by Alsaad et al. [40], confirming the potential of our nanocomposites for energy storage with minimal dissipation.

In this material, the optical transitions of electrons can be described by two additional significant parameters: the surface energy loss function (*SELF*), which characterizes electron transitions in thin materials, and the volume energy loss function (*VELF*), which characterizes electron transitions in bulk materials [47]. The *VELF* and *SELF* were calculated based on relations (9) and (10), respectively.

(9) $$SELF=\frac{\varepsilon^{\prime \prime }}{{\left(\varepsilon^{\prime \prime }+1\right)}^{2}+{\left(\varepsilon^{\prime }\right)}^{2}}$$

(10) $$VELF=\frac{\varepsilon^{\prime }}{{\left(\varepsilon^{\prime }\right)}^{2}+{\left(\varepsilon^{\prime \prime }\right)}^{2}}$$

**Figure 11:**
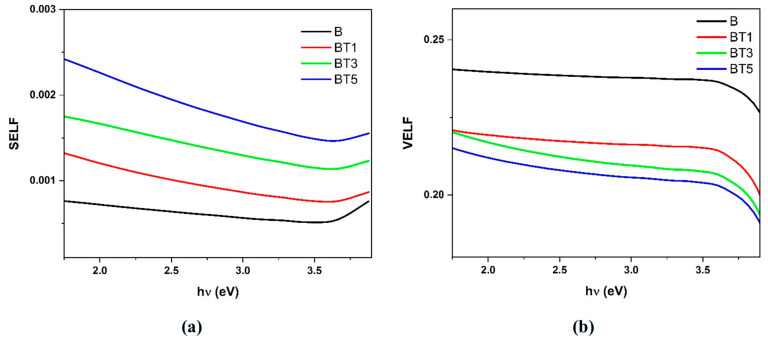
(**a**) Surface (*SELF*) and (**b**) volume (*VELF*) energy loss functions in relation to the photon energy of the different PVA-PVP-PAAm/TiO_2_ nanofibers.

Figure 11a,b illustrate the photon energy dependence of the *SELF* and *VELF* values of the different PVA-PVP-PAAm/TiO_2_ nanofibers. As the material composition changes due to TiO_2_ nanoparticles’ doping, altering the electron transition energy, both the *SELF* and *VELF* exhibit noticeable shifts. This can be attributed to the elastic strain energy, which originates from lattice mismatch and rapidly accumulates, progressively becoming the dominant influence. As a result, a direct interaction develops between the nanofibers and the surface [48].

### 3.5. Electrical Properties of Nanofibers

The electrical properties of nanofibers are an essential aspect of nanomaterial research [49]. The DC electrical conductivity ($\sigma_{\mathrm{DC}}$) was calculated using Equation (11), and the activation energy was determined using Equation (12).

(11) $$\sigma_{DC}=\frac{1}{\rho_{V}}=\frac{t}{R_{V}A_{s}}$$

where $\rho_{V}$ represents the volume resistivity, *t* is the sample thickness, $R_{V}$ denotes the volumetric electrical resistance, and $A_{s}$ is the surface area of the sample.

(12) $$\sigma_{DC}=\sigma_{0}\exp\left(-\frac{E_{a}}{k_{\beta }T}\right)$$

where $\sigma_{0}$ is the electrical conductivity extrapolated to absolute zero temperature, $E_{a}$ represents the activation energy, and $k_{\beta }$ denotes the Boltzmann constant.

**Figure 12:**
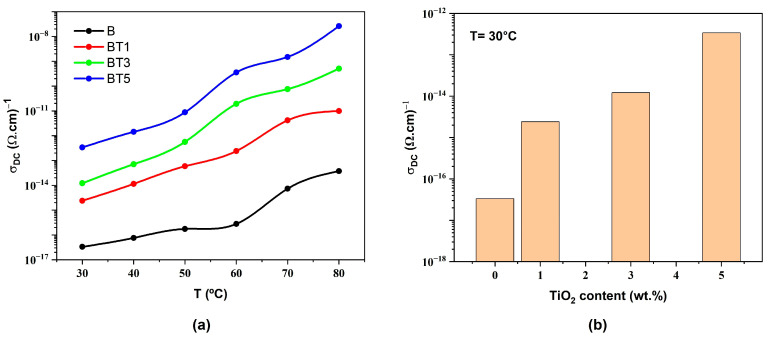
(**a**) Variation in DC electrical conductivity ($\sigma_{\mathrm{DC}}$) along with the temperature (T) and (**b**) Impact of TiO_2_ nanoparticle content on the room temperature DC electrical conductivity for PVA-PVP-PAAm/TiO_2_ nanofibers.

Figure 12a displays the temperature dependence of the electrical conductivity for all the samples, showing an increase in conductivity with rising temperature. The concentration of TiO_2_ in the PVA-PVP-PAAm blend influences the temperature-dependent behavior by enhancing polymer chain mobility and releasing trapped charges, which move via a hopping mechanism [50]. Elevated temperatures facilitate polymer chain motions, releasing trapped charges and boosting conductivity [51]. Consequently, the electrical conductivity is further enhanced due to the thermal activation of charge carriers, which gain sufficient kinetic energy to hop between trapping sites [52,53].

Figure 12b shows the room-temperature DC electrical conductivity of PVA-PVP-PAAm/TiO_2_ nanofibers as a function of TiO_2_ nanoparticle content. Increasing the TiO_2_ nanoparticle content to 5 wt.% significantly enhances conductivity by four orders of magnitude. This significant improvement is due to an increased number of charge carriers and the formation of an interconnected network of conductive pathways within the nanocomposite at higher TiO_2_ nanoparticle content. Additionally, TiO_2_ incorporation introduces energy levels within the bandgap of the polymer matrix, reducing the energy barrier and further enhancing conductivity [34,36].

**Figure 13:**
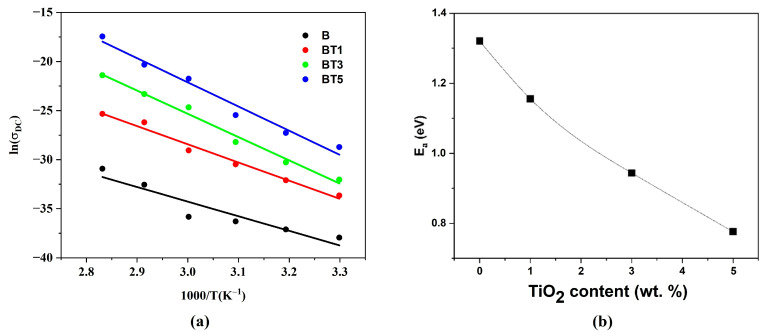
(**a**) Plot of ln ($\sigma_{\mathrm{DC}}$) as a function of the inverse absolute temperature and (**b**) variation in thermal activation energy ($E_{a}$) along with the content of TiO_2_ nanoparticles for PVA-PVP-PAAm/TiO_2_ nanofibers.

Figure 13a shows the plot of ln ($\sigma_{DC}$) versus (1000/T) for the PVA-PVP-PAAm/TiO_2_ nanofibers, from which the activation energy was calculated using Equation (12). As shown in Figure 13b, the thermal activation energy decreases with increasing TiO_2_ nanoparticle content. This reduction is attributed to the formation of localized energy levels within the bandgap, which act as trapping sites for charge carriers [54,55]. At 5 wt.% TiO_2_, the lower activation energy reflects the development of a continuous nanoparticle network within the nanofibers, facilitating charge transport [56]. These findings are consistent with reported thermal activation energies for PCL/TiO_2_ nanocomposites [36] and for PCL-CS/ZrO_2_ nanocomposites [57].

### 3.6. Biological Applications

#### 3.6.1. Antibacterial Activity

**Figure 14:**
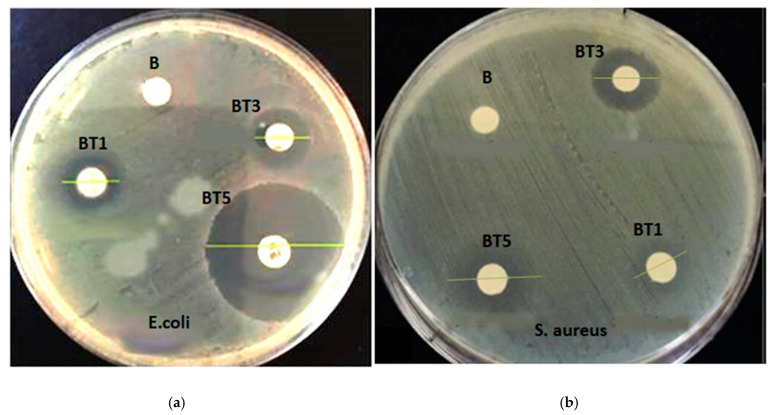
Antimicrobial activity against (**a**) Escherichia coli (*E. coli*) and (**b**) Staphylococcus aureus (*S. aureus*) bacteria. Inhibition zones are shown for: B (0 wt.% TiO_2_); BT1 (1 wt.% TiO_2_); BT3 (3 wt.% TiO_2_) and BT5 (5 wt.% TiO_2_) nanofibers.

Developing functional materials that inhibit bacterial growth while supporting healing processes is essential for biomedical applications [16]. In this study, the antibacterial properties of PVA-PVP-PAAm/TiO_2_ nanofibers were evaluated against Gram-negative Escherichia coli (*E. coli*, ATCC 35218) and Gram-positive Staphylococcus aureus (*S. aureus*, ATCC 29213) using a commonly used approach, which is the disk diffusion method to assess the antibacterial activity. After 24 h of incubation, inhibition zones were measured with a ruler to quantify antibacterial activity. The PVA-PVP-PAAm blend (sample B) showed no inhibition, whereas the PVA-PVP-PAAm/TiO_2_ nanofibers (BT1, BT3 and BT5) exhibited significant antibacterial properties, as shown in Figure 14 and Table 2. The diameter of the inhibition zones served as the metric for antibacterial activity, with ciprofloxacin (10 μg/mL) used as the positive control.

The PVA-PVP-PAAm/TiO_2_ nanofibers demonstrated larger inhibition zones against *E. coli* compared to *S. aureus*. For instance, sample BT5 (5 wt.% TiO_2_) achieved inhibition zones of 18.2 ± 0.2 mm for *E. coli* and 11.6 ± 0.2 mm for *S. aureus* (Table 2). These differences arise from variations in bacterial cell wall structures. Gram-negative bacteria, such as *E. coli*, possess a double-layered membrane and a thin peptidoglycan layer, which may limit TiO_2_ nanoparticle penetration but enhance electrostatic attraction between positively charged Ti^4+^ ions and the negatively charged cell walls, improving antibacterial efficacy [58,59]. In contrast, the thick peptidoglycan layer of Gram-positive *S. aureus* may reduce nanoparticle uptake, resulting in smaller inhibition zones [58]. The PVP-PAAm/TiO_2_ nanofibers exhibit significant antimicrobial properties, making them promising candidates for high-safety and hygiene applications in medical devices, such as antimicrobial coatings and wound dressing [60]. Their combined antioxidant and antibacterial activities offer several benefits, enabling advancements in food preservation, wound healing, and medical device coating by simultaneously preventing oxidative degradation and inhibiting microbial growth.

#### 3.6.2. Antioxidant Activity

The 1,1-diphenyl-2-picrylhydrazil (DPPH) radical is a stable free radical widely used to assess antioxidant capacity through spectrophotometric measurements. Its intense violet color, attributed to π–π* transitions with a maximum absorption at 517 nm in ethanol, fades to pale yellow upon reduction to hydrazine (DPPH-H) by hydrogen atom donation from antioxidants [58,59,60,61]. The radical’s stability arises from steric crowding around the nitrogen atom and the “push-pull” effect between electron-donating diphenylamino and electron-accepting picryl groups, preventing dimerization and enabling its use in electron paramagnetic resonance spectroscopy and polymer chemistry. DPPH, which is soluble in polar organic solvents such as methanol but nearly insoluble in water, selectively reacts with hydrogen donors at its nitrogen atom. This characteristic, combined with its short reaction times and adherence to Beer-Lambert’s law, makes it an effective tool for evaluating the antioxidant activity of phenolic compounds, herbal extracts, and food ingredients.

**Figure 15:**
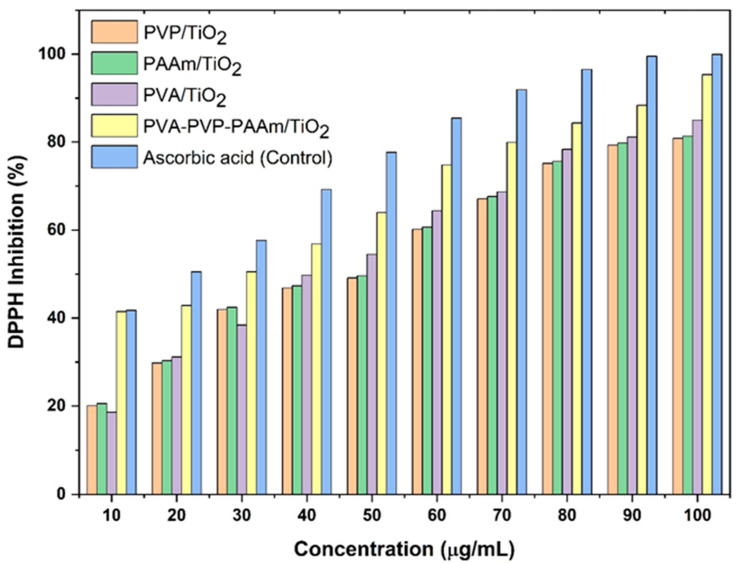
DPPH inhibition percentage for various nanofiber samples (PVP/TiO_2_, PAAM/TiO_2_, and PVA-PVP-PAAm/TiO_2_), each containing 5 wt.% TiO_2_. Ascorbic acid served as the reference control.

The antioxidant activity of the electrospun nanofibers was evaluated using the DPPH assay. By immobilizing the DPPH radical within the nanofiber matrix, the nanofibers’ ability to scavenge free radicals through hydrogen donation and electron quenching was directly evaluated. The TiO_2_-containing nanofibers (PVP/TiO_2_, PAAm/TiO_2_, PVA/TiO_2_, and PVA-PVP-PAAm/TiO_2_) exhibited dose-dependent improvement in the scavenging capacity, as shown in Figure 15. Notably, the PVA-PVP-PAAm/TiO_2_ nanofiber demonstrated the highest performance, achieving a DPPH of 95.32% at 100 µg/mL, comparable to that of ascorbic acid (99.94%, *p*-value < 0.005). During inflammation, excessive reactive oxygen species (ROS) production can degrade lipids and nucleic acids, leading to biological damage and cell death [62]. Antioxidants mitigate these effects by supporting cellular metabolism [63]. Numerous studies have confirmed nanomaterials as significant sources of antioxidant activity [64,65]. As the concentration of PVA-PVP-PAAm/TiO_2_ nanofibrous mats increased, DPPH free radical scavenging also significantly improved, aligning with findings by Venkatappa et al. [16], who reported enhanced DPPH inhibition with TiO_2_ nanoparticles.

## 4. Conclusions

This study successfully fabricated and characterized electrospun PVA-PVP-PAAm/TiO_2_ nanofibers, demonstrating substantial enhancements in structural, optical, electrical, and biological properties. The incorporation of TiO_2_ nanoparticles (0–5 wt.%) increased nanofiber diameters from 21.5 nm to 35.1 nm and improved crystallinity. FTIR spectra confirmed stronger hydrogen bonding, evidenced by the shifts in O–H stretching vibrations near ~3200 cm^−1^, indicating robust TiO_2_–polymer interactions. The optical band gap decreased from 3.575 eV to 3.320 eV, accompanied by an increase in the refractive index, suggesting potential applications in optoelectronic devices. Electrical conductivity at room temperature improved markedly from 3 × 10^−17^ (Ω·cm)^−1^ for 0 wt.% TiO_2_ to 3 × 10^−13^ (Ω·cm)^−1^ for 5 wt.% TiO_2_, attributed to interconnected conductive pathways and reduced activation energies. Nanofibers containing 5 wt.% TiO_2_ exhibited superior antibacterial efficacy, with inhibition zones of 18.2 ± 0.2 mm against *E. coli* and 11.6 ± 0.2 mm against *S. aureus*, as well as exceptional antioxidant activity, achieving 95.32% DPPH inhibition, comparable to ascorbic acid. These enhancements, driven by synergistic TiO_2_–polymer interactions, confer additional functionalities, such as UV shielding and radical scavenging. Collectively, the PVA-PVP-PAAm/TiO_2_ nanocomposites exhibit strong potential for real-world applications, including antimicrobial coatings and wound dressings for healthcare, food solutions for industry stakeholders, and UV-shielding materials for environmental sensors and optoelectronic devices.

## Figures and Tables

**Table 1:** Optical band gap and Urbach energy for the different PVA-PVP-PAAm/TiO_2_ nanofibers.

Sample	$Allowed\;E_{g\;indir.}^{opt}\;\left(\mathrm{eV}\right)$ Tauc Relation	$E_{ASF}^{opt}\;\left(\mathrm{eV}\right)$ ASF Method	$E_{U}\;\left(\mathrm{eV}\right)$
B	3.575	3.499	0.208
BT1	3.501	3.429	0.265
BT3	3.421	3.391	0.417
BT5	3.320	3.276	0.507

**Table 2:** Diameter of the inhibition zones on *S. aureus* and *E. coli* bacteria with PVA-PVP-PAAm/TiO_2_ nanofibers.

Sample	*E. coli.* Diameter of Inhibitory Zone (mm)	*S. aureus* Diameter of Inhibitory Zone (mm)
B	0	0
BT1	7.5 ± 0.2	7.3 ± 0.2
BT3	8.2 ± 0.2	7.7 ± 0.2
BT5	18.2 ± 0.2	11.6 ± 0.2

## Data Availability

The original contributions presented in this study are included in the article. Further inquiries can be directed to the corresponding author.
